# MiR‐766‐3p Inhibit the Proliferation, Stemness, and Cell Cycle of Pancreatic Cancer Cells Through the MAPK/ERK Signaling Pathway

**DOI:** 10.1002/mgg3.70049

**Published:** 2024-12-18

**Authors:** Zhipeng Quan, Ziwei Yin, Yuelin Huang, Xuemei Huang, Hao Huang, Qingrong Mo, Jianhua Gong, Lingyun Liu, Yi Zhou, Yaqun Yu

**Affiliations:** ^1^ Department of Hepatobiliary and Pancreatic Surgery Affiliated Hospital of Guilin Medical University Guilin Guangxi China; ^2^ Key Laboratory of Early Prevention and Treatment for Regional High Frequency Tumor (Guangxi Medical University) Ministry of Education Nanning Guangxi China; ^3^ Department of Oncology, Third Xiangya Hospital Central South University Changsha China; ^4^ Department of General Surgery, Second Xiangya Hospital Central South University Changsha China; ^5^ Health Management Center Affiliated Hospital of Guilin Medical University Guilin Guangxi China; ^6^ Department of Hepatobiliary and Pancreatic Surgery The First College of Clinical Medical Science (China Three Gorges University) Yichang China; ^7^ Department of Hepatobiliary and Pancreatic Surgery Yichang Central People's Hospital Yichang Hubei China; ^8^ Guangxi Key Laboratory of Molecular Medicine in Liver Injury and Repair The Affiliated Hospital of Guilin Medical University Guilin Guangxi China

**Keywords:** *MAPK/ERK*, *MAPK1*, *MIR‐766‐3P*, pancreatic cancer

## Abstract

**Background:**

As a commonly identified cancer in clinics, pancreatic cancer (PC) has poor prognostic outcomes. This work focused on clarifying the association between *MIR‐766‐3P* expression and PC development and progression, as well as the possible role as a biomarker in PC.

**Methods:**

*MIR‐766‐3P* expression within the human PC cells and samples was measured through miRNA RT‐PCR. The gene levels regulated by *MIR‐766‐3P* were analyzed through western blot (WB) and qRT‐PCR. To analyze whether *MIR‐766‐3P* was of certain significance in in vitro and in vivo PC cell proliferation, stemness, and cell cycle progression, the gain/loss‐of‐function assays were performed. Bioinformatics, RNA sequencing (RNA‐seq), and luciferase reporter assay were conducted for exploring regulatory role of *MIR‐766‐3P*/*MAPK1*/*MAPK/ERK* signal axis in PC.

**Result:**

In comparison with the normal controls, *MIR‐766‐3P* expression markedly decreased the tissues and cells of PC. Furthermore, *MIR‐766‐3P* could remarkably inhibit the proliferation, stemness, cell cycle progression, and development of PC. The analyses using RNA‐seq, and dual‐luciferase examination showed that *MIR‐766‐3P* could directly target mitogen‐activated protein kinase 1 (*MAPK1*). According to Gene Ontology (GO) as well as Kyoto Encyclopedia of Genes and Genomes (KEGG) analysis, *MIR‐766‐3P* could affect PC malignant phenotype by *MAPK1* and the regulation of the *MAPK/ERK*‐related pathway.

**Conclusion:**

*MIR‐766‐3P* has a certain impact on PC malignant phenotype through combining with *MAPK1* while regulating *MAPK/ERK*‐related pathway in vitro and in vivo.

## Graphical abstract

Diagram of miR‐766‐3P's role in pancreatic cancer (PC) and its mechanism. miR‐766‐3P targeting MAPK1 and activating the MAPK/ERK pathway to regulate the malignant phenotype of PC.

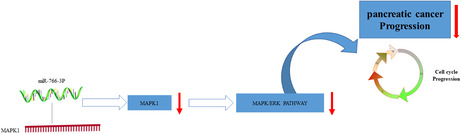

## 1 Introduction

Pancreatic cancer (PC) is more commonly encountered in the general population than in clinical settings. Currently, surgical treatment is still an optimal curative modality for PC, but only 20% of patients can be surgically treated due to the inaccessibility of effective PC screening, most cases (30%–35%) are at the late stage at diagnosis or developing distant metastases (DM, 50%–55%). This also leads to very poor survival and equal death cases to diagnosed cases. PC may surpass breast cancer (BC) by 2025 to be the third major cause inducing cancer‐associated mortality (Park, Chawla, and O'Reilly 2021; Sung et al. 2021; Ferlay, Partensky, and Bray 2016). Therefore, it is urgently needed to find efficient molecular markers and novel therapeutic targets for PC treatment and therapy.

MicroRNAs (miRNAs), the small endogenous noncoding RNAs (ncRNAs) that contain 22–28 nucleotides, have been found to directly interact with the target gene 3'UTR for regulating gene expression at the mRNA level. Therefore, miRNAs play roles of tumor promoters or suppressors, as well as play important effects on cancer development and progression through several pathways (Bahubeshi, Tischkowitz, and Foulkes 2011; Lee, Feinbaum, and Ambros 1993; Wightman, Ha, and Ruvkun 1993; Kasinski and Slack 2011; Rupaimoole et al. 2016). As revealed by numerous studies, miRNAs have crucial effects on different cell events, like cell proliferation, stem maintenance, cell cycle, apoptosis, migration, invasion, and differentiation.

*MIR‐766‐3P* has been confirmed to be a suppressor of several tumors and is significantly downregulated in various tumors, such as bladder cancer (Zhou, Zhu, and Man 2020), kidney cancer (Chen et al. 2017), liver cancer (Liu et al. 2019), and colorectal cancer (He et al. 2022). Nonetheless, its effect on PC initiation, migration, and progression is largely uninvestigated, and its underlying mechanisms in regulating tumorigenesis and progression remain largely unclear.

Mitogen‐activated protein kinases (*MAPK*, OMIM: 603296) belong to the Ser/Thr kinase family (Cargnello and Roux 2011). They are related to tumor cell growth, development, and differentiation when activated and may serve as the therapeutic target (McCubrey et al. 2007; Chong, Vikis, and Guan 2003; Najafi, Ahmadi, and Mortezaee 2019; Montagut and Settleman 2009). Recent studies have shown that *MAPK1* (ERK2, OMIM: 176948) regulates hepatocarcinogenesis and development (Liu et al. 2021). However, its effect on PC remains largely unclear.

In our present study, we utilized RNA‐seq analyses to identify *MAPK1* as a target of *MIR‐766‐3P*. Furthermore, we found that the *MIR‐766‐3P/MAPK1* axis regulates cell proliferation, cell cycle progression, and stem cell maintenance in PC by modulating the *MAPK/ERK* signaling pathway.

## 2 Materials and Methods

### 2.1 Ethical Compliance

The study has been approved by the Ethics Committee of the Affiliated Hospital of Guilin Medical University (No. KJTLL202140) and the approval date is September 13, 2021. All participants in the study have provided appropriate informed consent as required by the ethics committee. The animal experiments have been approved by the Laboratory Animal Ethics Committee of Guilin Medical University (No. GLMC202105162), and the approval date is May 16, 2021.

### 2.2 PC Tissues

Our study protocols gained approval from Ethics Committee of Affiliated Hospital of Guilin Medical College. This work was carried out following Helsinki declaration. Participants signed informed consent before the initiation of this study. The PC samples (*n* = 50) and matched adjacent tissue samples (*n* = 50) were collected from the PC patients receiving radical surgical treatment. After removing of the tumor in operating room, tumor tissue was harvested at once and immersed within liquid nitrogen, followed by preservation under −80°C.

### 2.3 Cells and Cell Culture

Human normal HPDE6C7 pancreatic cells and six PC cells (PANC‐1, ASPC‐1, SW1990, Capan‐2, BXPC‐3, and MiaPaca‐2) utilized in the present work were acquired from American type culture collection (ATCC; Manassas, VA, USA). After resuscitation, cells were cultured within 1% penicillin/streptomycin (PS) and 10% fetal bovine serum (FBS, GIGCO, USA)‐contained DMEM. The cells were grown within an incubator with 100% humidity under 37°C with 5% CO_2_. Cell passage was conducted at 1‐ to 2‐day intervals to maintain logarithmic growth.

### 2.4 Immunohistochemical (IHC) Analysis

For immunohistochemical (IHC) analysis, 10% formalin was added to fix PC tissues, followed by cutting in 5‐μm sections, xylene dewaxing and gradient hydration using ethanol. Each section was later heated in 0.01 M citric acid antigen retrieval solution (pH 6.0) for 20 min to retrieve the antigen before the IHC staining. 3% H_2_O_2_ was added to block the endogenous peroxidase activity for a 20‐min period under ambient temperature. Subsequently, to block nonspecific binding, the sections were blocked using goat serum for a 1‐h period. Then, each section was subject to overnight primary antibody incubation under 4°C. Following PBS rinsing thrice, horseradish peroxidase (HRP)‐labeled secondary antibodies were added to incubate sections for a 1‐h period under ambient temperature. Following rinsing by PBS thrice, the HRP 3,3′‐diaminobenzidine (DAB) kit (Thermo Fisher Scientific, Waltham, Ma, USA) was used for color development. After hematoxylin counterstaining, neutral resin was added for section sealing. The Olympus ×71 inverted microscope (Olympus Corp., Tokyo, Japan) was used to obtain images. Finally, a positively stained cell proportion was combined with the corresponding intensity to analyze staining. Antibody information used is shown in Tables S1.

### 2.5 Western Blot (WB) Assay

To extract total cellular and tissue proteins, the RIPA lysis buffer (Solarbio, China) was used in line with specific protocols. A BCA Protein Quantification Kit (Thermofisher Scientific, USA) was employed for determining protein contents. Subsequently, 4.0 × SDS loading buffer (Solarbio, China) was added, followed by 10‐min sample denaturation at 98°C before electrophoresis. Then, the samples were separated using 10% PAGE‐gel, followed by transfer onto the PVDF membrane (biorad, Shanghai, China). Next, after 2‐h blocking by 5% defatted milk, membranes were washed by TBST thrice (5 min each). Later, primary antibodies were added to incubate proteins overnight under 4°C. Following rinsing by TBST, membranes were subject to 2‐h secondary antibody incubation at room temperature. Enhanced chemiluminescence ECL reagents (Affinity, China) were added to visualize protein bands. ImageJ software was utilized to quantify band intensity. Each experiment was conducted three times. Antibody information used is shown in Table S1.

### 2.6 qRT‐PCR and miRNA RT‐PCR

To extract total tissue or cellular RNA, the Trizol reagent (Invitrogen, USA) was used following specific protocols. Using agarose gel electrophoresis, the integrity and purity of the RNA samples were verified. The goldenstartm RT6 cDNA synthesis kit was used for reverse transcription (RT) following specific instructions. To detect mature *MIR‐766‐3P*, we polyadenylated the total RNA using poly (A) polymerase (Ambion, Austin, TX, USA). Total poly (A)–tailed RNA, reverse transcription primers (Table S2), and ImPro‐II reverse transcriptase (Promega, Fitch‐burg, WI, USA) were used for reverse transcription as per the manufacturer's instructions. Later, SYBR Green Master (Bio‐RAD, USA) was utilized for qPCR. Furthermore, primescript RT reagent kit (Takara, Dalian, China) was used to synthesize cDNA using extracted total RNA prior to qRT‐PCR analysis. The *U6* or *GAPDH* expression served as endogenous controls. Using a 2^−ΔΔCT^ approach, relative gene levels were quantified. This study utilized MAPK1, MIR‐766‐3P, GAPDH, and U6 primers, all of which were purchased from Tsingke Biotechnology (Tsingke, Beijing, China). Table S2 displays primer sequences utilized in this work.

### 2.7 Vector Construction, Lentivirus Production, and Cell Transfection

The plv‐hsa‐*MIR‐766‐3P*‐sponge inhibitor vector (*MIR‐766‐3P* inhibitor#1, inhibitor#2), plv‐hsa‐*MIR‐766‐3P*‐premicrorna lentiviral vector (*MIR‐766‐3P* mimics), together with the vector that contained *MAPK1* sequence (*MAPK1*) was synthesized by Tsingke Biotechnology (Tsingke, Beijing, China). PC cells were transfected following specific instructions with Lipofectamine 3000 (Invitrogen, CA, USA). The study was conducted using the *MAPK1* gene sequence (NCBI Reference Sequence: NM_002745.5).

### 2.8 Cell Viability Assay

This work carried out cell counting kit‐8 assay (CCK‐8, Dojindo, Tokyo, Japan) for detecting cell viability in accordance with specific protocols. Thereafter, the present work cultured transfected PC cells (2000/well) in 96‐well plates as previously described. After 1‐, 2‐, 3‐, and 4‐day incubation, cells were incubated by CCK‐8 solution (10 μL) for a 2‐h period, and then the microplate reader was employed for measuring absorbance at a wavelength of 450 nm.

### 2.9 Clone Formation Assay

This work inoculated PC cells (500/well) in 6‐well plates under the same conditions mentioned above for 14 days. When visible colonies could be observed with the naked eye, cells were rinsed in each well with PBS three times, followed by 30 min fixation by 4% paraformaldehyde (PFA), then staining by 0.5% crystal violet (Solarbio, China). A scanner (Microtek, China) was utilized to photograph colonies, followed by manual colony counting.

### 2.10 Spheroid Formation Assay

The cells were suspended, seeded (1000/mL) in 6‐well ultralow attachment plates (Corning, NY, USA), and cultured for 6 days to obtain sphere 24 h after transfection. The total number and diameter of the spheres were counted under a microscope (Olympus, Beijing, China). Subsequently, sphere‐forming quantity/inoculated cell count (1000/well within the 24‐well plate) × 100% was applied for calculating sphere formation rate. Furthermore, the spheroid volume was determined by formula *V* = (4/3)πR^3^. The experiment was conducted thrice independently.

### 2.11 Cell Cycle Progression

After transfection, cells were digested with EDTA‐free trypsin and centrifuged at 1200 g for a 5‐min period according to specific protocols (keygen bio) and twice rinsing with PBS. After centrifugation again, the supernatant was removed, and cells were fixed by 70% alcohol under 4°C overnight. Cells were rinsed with PBS twice, followed by addition of PI (500 UL) to incubate for a 60‐min period in dark under ambient temperature. A BD facscanto II (BD Biosciences, USA) was employed for cell cycle analysis.

### 2.12 Luciferase Reporter Assay

For obtaining *MIR‐766‐3P* ‘s possible binding sites with the target, the targetscan database (Bartel laboratory, Whitehead Institute for Biomedical Research, Cambridge, Ma, USA) was used. And the above results were verified by luciferase reporter gene testing. Briefly, downstream luciferase reporter gene was cloned in wildtype (WT) or mutant (MUT) *MAPK1* 3'‐UTR sequence within the luciferase vector (Invitrogen). Furthermore, cells were subject to cotransfection using *MIR‐766‐3P* mimics with WT or MUT *MAPK1*‐3'UTR contained pgl3 vector. Besides, cells were cotransfected using Lipofectamine 3000 (Invitrogen) and miR‐NC or *MIR‐766‐3P* mimics and vector according to specific instructions. Luciferase activity was detected by a dual‐luciferase assay system (Promega), which was normalized by coexpressed Renilla luciferase.

### 2.13 Xenograft Tumor Assays

This work gained approval from Animal Experimental Ethics Committee of Guilin Medical University (approval no. GLMC202105162). The animals used were treated humanely. The male balb/c nude mice (6‐week‐old) were provided by the experimental animal center of Guilin Medical University. The cells were subjected to trypsin digestion, resuspended in precooled phosphate‐buffered saline (PBS), add Matrigel (Sigma‐Aldrich) to accelerate the formation of tumor spheroids, with a ratio of Matrigel to PBS of 1:1, and the concentration was adjusted to 1 × 10^7^ cells/mL. Each nude mouse was subcutaneously injected with 0.1 mL of suspension containing 1 × 10^6^ cells overexpressing *MIR‐766‐3P* and negative control cells. Tumor formation was monitored over a period of 6 weeks.

### 2.14 Transcriptome Sequencing

The QIAGEN RNeasy Mini Kit (QIAGEN, Germany) was used to isolate total RNA from cell samples, following the manufacturer's instructions. The RNA was then assessed for quality and quantified to ensure it was suitable for sequencing. RNA sequencing was conducted using the Illumina NovaSeq 6000. Data normalization was achieved with Affymetrix's Expression Console software using the RMA algorithm. Custom Perl scripts were used for adapter removal and quality control. A *p* value of 0.05 was set for significant differential expression, with a four‐fold change as the threshold for gene upregulation and downregulation. We analyzed differentially expressed genes using the Gene Ontology (GO; http://www.geneontology.org) and the Kyoto Encyclopedia of Genes and Genomes (KEGG; http://genome.jp/kegg).

### 2.15 Statistical Analysis

This work applied SPSS 21.0 software in implementing each assay and data analysis. Each assay was carried out three times independently. All results were represented by mean ± SD. The correlations of *MIR‐766‐3P* levels with clinicopathological characteristics were evaluated using the chi‐squared test. Spearman correlation coefficients (SCC) were calculated for the analysis of the correlation of *MIR‐766‐3P* with *MAPK1*. Survival analysis was completed using Kaplan–Meier analysis, while the log‐rank test was utilized to predict different survival probabilities. *p* < 0.05 stood for statistical significance. “*” means “*p* < 0.05”, “**” means “*p* < 0.01”, “***” means “*p* < 0.001”.

## 3 Results

### 3.1 Decreased MIR‐766‐3P Expression Within PC Cells and Tissues Predicts Poor Prognostic Outcome

**FIGURE 1:**
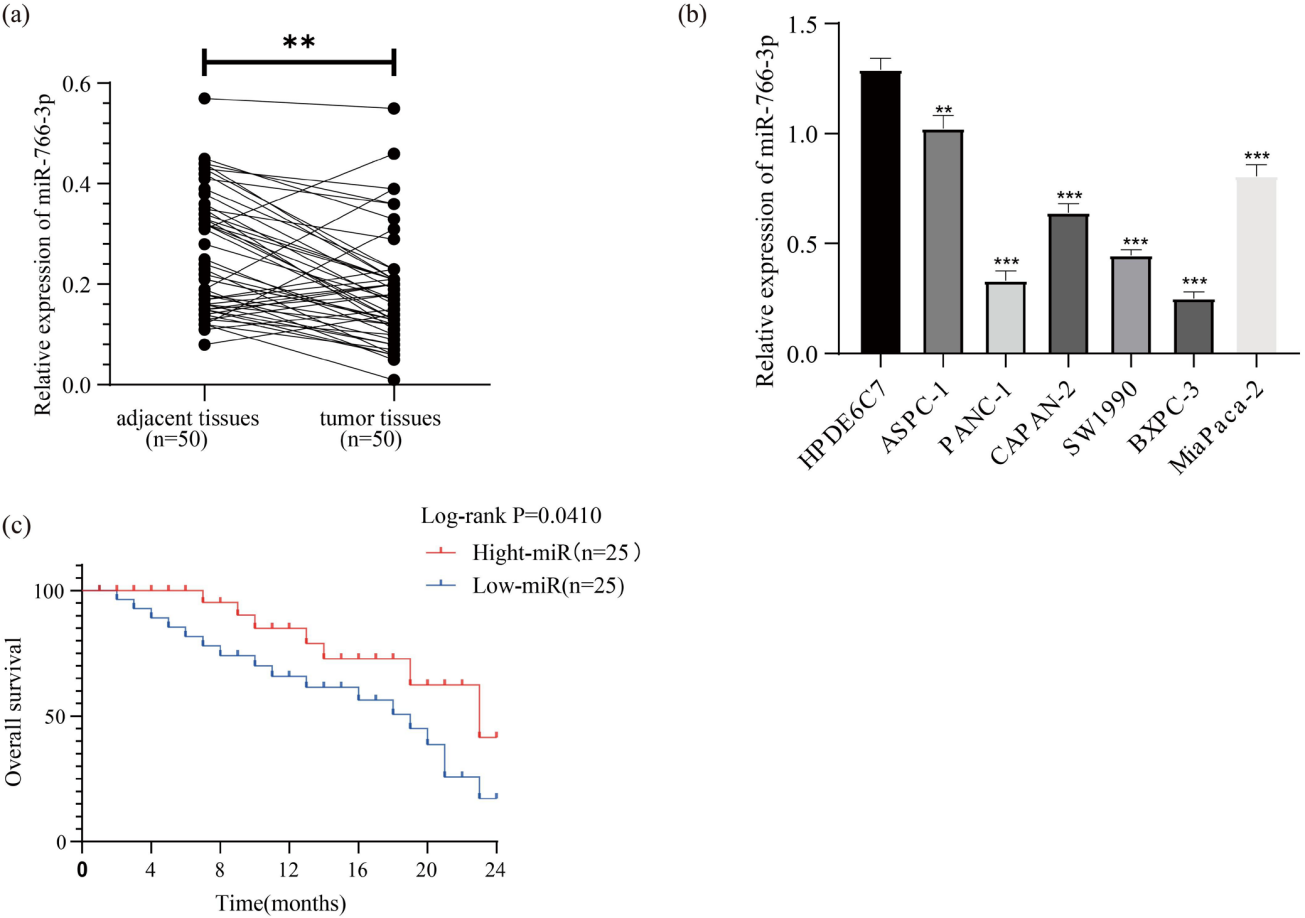
miR‐766‐3p levels within PC tissues and PC cells, and its downregulation predicts dismal prognostic outcome. (a) miR‐766‐3p levels within 50 PC and 50 corresponding noncarcinoma samples were measured through miRNA RT‐PCR (*n* = 50). (b) miR‐766‐3p levels within PC cell lines and normal pancreatic cell HPDE5C7. (c) Kaplan–Meier analysis on the relation of miR‐766‐3p level with PC prognostic outcome among 50 cases (*n* = 3, **p < 0.01, ***p < 0.001).

For detecting *MIR‐766‐3P* levels within PC clinical tissues, 50 PC tissue samples and another 50 corresponding adjacent tissue samples were collected. In comparison with the adjacent tissue samples, significantly decreased *MIR‐766‐3P* levels could be observed within the PC tissues using miRNA RT‐qPCR (Figure 1a). These results were also supported by the data obtained from human HPDE6C7 pancreatic cell and PC cell lines in vitro (Figure 1b). For exploring the relationship of *MIR‐766‐3P* expression with PC prognosis, we classified the patients as high or low expression group according to median *MIR‐766‐3P* level. According the results for the Kaplan–Meier survival analysis, high‐*MIR‐766‐3P* expression group had superior prognostic outcome to low‐*MIR‐766‐3P* expression group (Figure 1c). Based on these findings, *MIR‐766‐3P* was speculated to have an essential effect on PC genesis and progression.

### 3.2 Effect of MIR‐766‐3P on PC Cell Proliferation, Stemness, and Cell Cycle Progression

**FIGURE 2:**
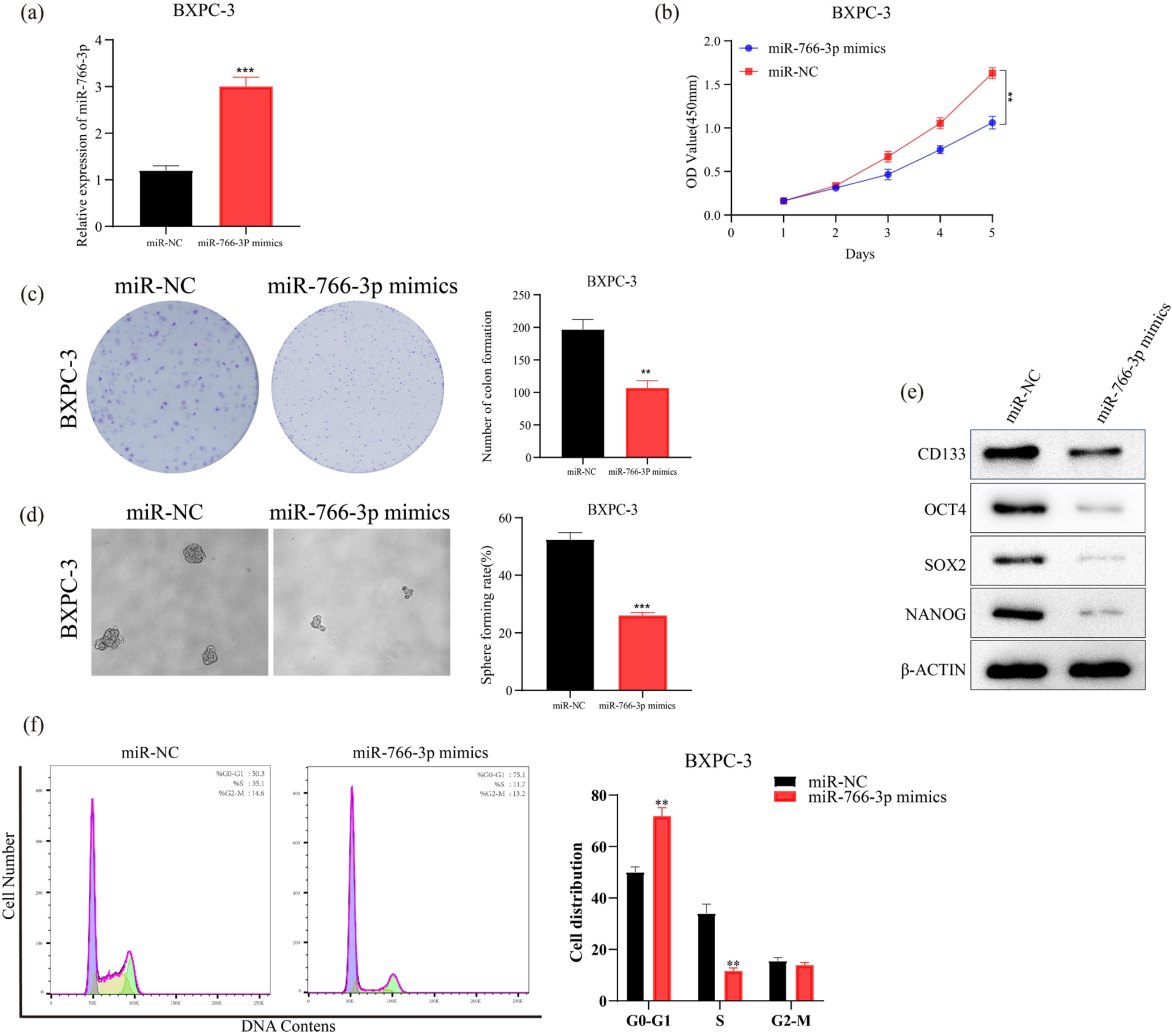
miR‐766‐3p upregulation could inhibit the proliferation, decreases the spheroid forming ability, and regulates the cell cycle (a) qRT‐PCR was conducted for detecting miR‐766‐3p expression within BXPC‐3 cells 48 h following miR‐766‐3p mimics transfection. (b) As suggested by CCK‐8 assay, miR‐766‐3p mimics transfection suppressed BXPC‐3 cell growth. (c) miR‐766‐3p overexpression suppressed in vitro clone‐forming ability in BXPC‐3 cells. (d) Statistical analyses of the spheroid formation in BXPC‐3 cells after miR‐766‐3p mimics transfection. Typical images presenting spheroid formation within such cells are presented (100 μm). (e) WB assay was conducted to measure stemness‐associated gene levels within BXPC‐3 cells after miR‐766‐3p mimics transfection. (f) Analysis of cell cycle in BXPC‐3 cells after miR‐766‐3p mimics transfection (*n* = 3, **p < 0.01, ***p < 0.001).

**FIGURE 3:**
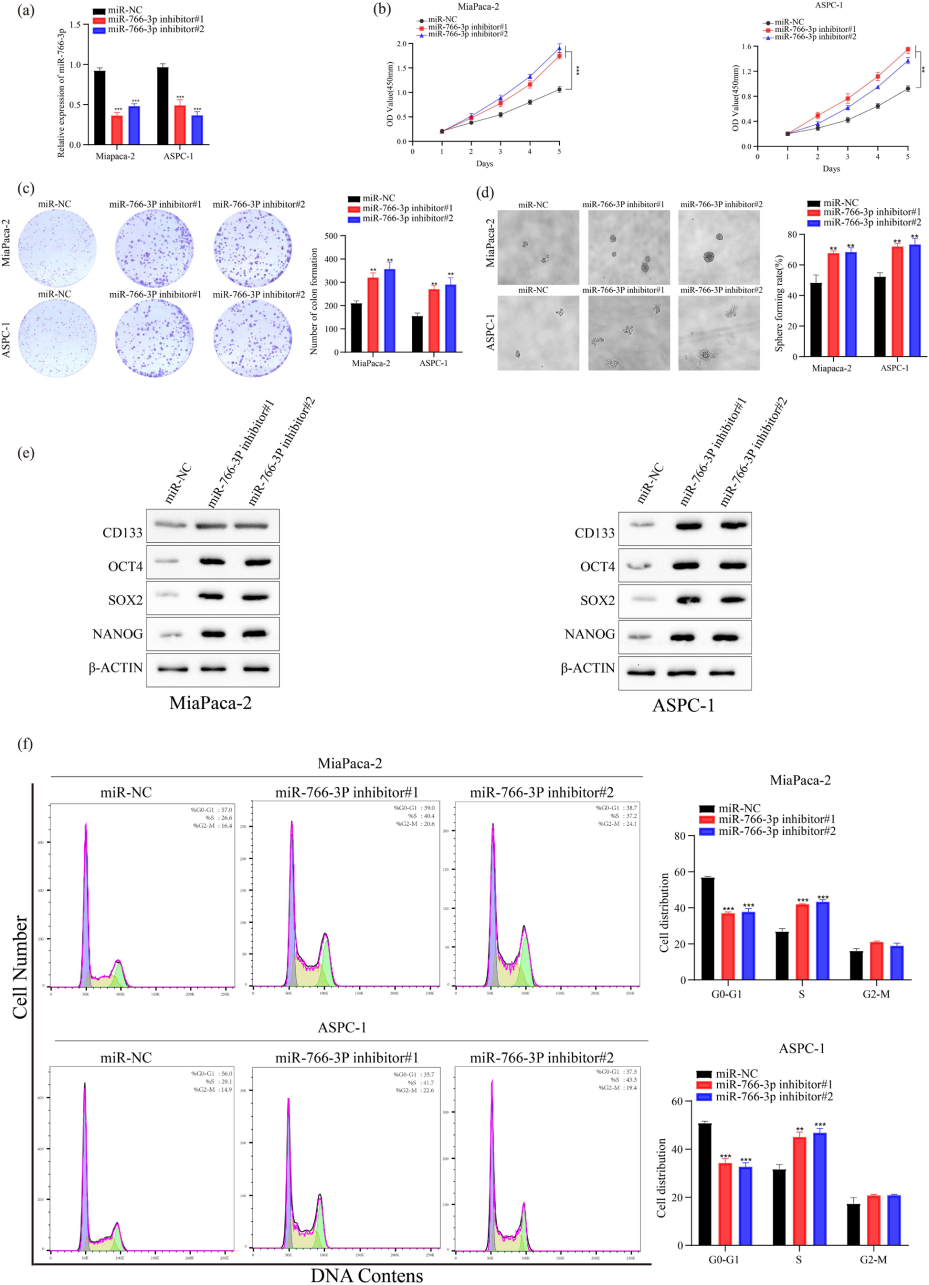
miR‐766‐3p downregulation could promote the proliferation, spheroid forming ability and regulates the cell cycle. (a) qRT‐PCR was conducted for detecting miR‐766‐3p expression within MiaPaca‐2 and ASPC‐1 cells 48 h following miR‐766‐3p inhibitor transfection. (b) As suggested by CCK‐8 assay, miR‐766‐3p inhibitor transfection promoted MiaPaca‐2 and ASPC‐1 cell growth. (c) Low expression miR‐766‐3p promote in vitro clone‐forming abilities of MiaPaca‐2 and ASPC‐1 cells. (d) Statistical analyses of the spheroid forming rate of MiaPaca‐2 and ASPC‐1 cells after miR‐766‐3p inhibitor transfection (100 μm). (e) WB assay was conducted to measure stemness‐associated gene levels within MiaPaca‐2 and ASPC‐1 cells after miR‐766‐3p inhibitor transfection. (f) Analysis of cell cycle in MiaPaca‐2 and ASPC‐1 cells after miR‐766‐3p inhibitor transfection (*n* = 3, **p < 0.01, ***p < 0.001).

Based on *MIR‐766‐3P* expression (Figure 1b), PC cell lines (MiaPaca‐2, BXPC‐3, and ASPC‐1) were selected in our further experiments. These three cell lines have better typicality and representativeness. Among six PC cell lines in this study, the MiaPaca‐2 and ASPC‐1 cell lines have high‐MiR‐766‐3p expression, so we performed MiR‐766‐3p knockdown. In contrast, the BXPC‐3 cell line has low‐MiR‐766‐3p levels, leading us to conduct overexpression treatment. Using exogenously transfecting *MIR‐766‐3P* inhibitor or mimics, the *MIR‐766‐3P* overexpressed BXPC‐3 cells, and *MIR‐766‐3P* silenced MiaPaca‐2 and ASPC‐1 cells were established. Transfection efficiency was verified by miRNA RT‐PCR. The significantly elevated and reduced *MIR‐766‐3P* expression was detected within BXPC‐3 cells and MiaPaca‐2 and ASPC‐1 cells, respectively, in comparison with their corresponding controls (Figures 2a and 3a).

The remarkably suppressed and enhanced cell growth were detected within the *MIR‐766‐3P* overexpression (Figure 2b) and silencing (Figure 3b) cells, respectively by the detection of CCK‐8 together with plate clone‐forming assay. Furthermore, *MIR‐766‐3P* overexpression in BXPC‐3 could significantly suppress the formation of cell colony (Figure 2c), while the suppression of *MIR‐766‐3P* expression showed an opposite impact on PC cells' colony formation (Figure 3c). Collectively, *MIR‐766‐3P* significantly participated in PC cell growth and clonogenic abilities.

Next, we analyzed *MIR‐766‐3P*'s impact on PC cell stemness. In comparison with controls, *MIR‐766‐3P* overexpression cells exhibited remarkably decreased cell spheroid formation (Figure 2d). On the contrary, the PC cells spheroid formation was significantly enhanced after *MIR‐766‐3P* suppression (Figure 3d). Subsequently, this work examined *MIR‐766‐3P*'s impact on cancer stem cell (CSC) marker levels and genes associated with CSC, like *OCT4*, *CD133*, *SOX2*, and *NANOG*. Obviously reduced and increased levels of the above four factors were observed within cells with *MIR‐766‐3P* overexpression (Figure 2e) and suppression (Figure 3e), respectively, compared with the control cells. Based on these findings, we concluded that *MIR‐766‐3P* maintained PC cell stemness. *MIR‐766‐3P* overexpression reduced PC cell stemness.

Thereafter, this work examined how *MIR‐766‐3P* affected the cell cycle through flow cytometry (FCM). As a result, compared with the control cells, more *MIR‐766‐3P* overexpressed cells at G0/G1 phase (Figure 2f) whereas less *MIR‐766‐3P* silenced cells at G0/G1 phase were observed (Figure 3f), suggesting *MIR‐766‐3P*'s regulation on the cell cycle progression in PC cells. Taken together, *MIR‐766‐3P* overexpression significantly blocked cell cycle arrest at G0/G1 phase, thus impairing PC cell development.

### 3.3 Role of MIR‐766‐3P in MAPK1

**FIGURE 4:**
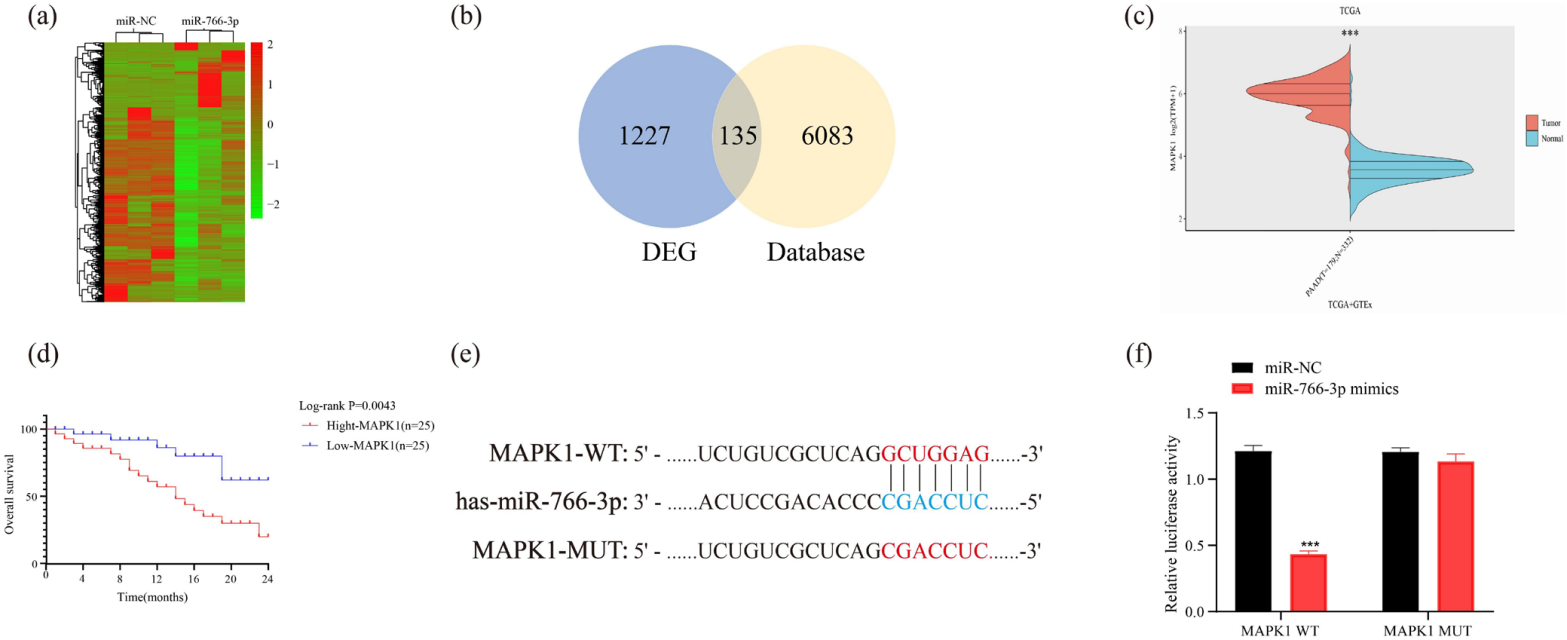
miR‐766‐3p suppresses MAPK1 level through the direct targeting of putative binding site of MAPK1 3'‐UTR. (a) DEGs supervised hierarchical clustering following miR‐766‐3p overexpression within BXPC‐3 cells. (b) By Combining the web‐based prediction database and screened differentially expressed genes, altogether 135 possible genes were obtained from those selected DEGs. (c) expression of MAPK1 in TCGA and GTEX databases. (d) Kaplan–Meier analysis on relation of MAPK1 level with PC prognostic outcome of 50 cases. (e) Bioinformatics prediction approaches were adopted for detecting possible binding sites in miR‐766‐3P to MAPK1 3′‐UTR. (f) Role of miR‐766‐3P in p‐MAPK1‐wt and p‐mut‐MAPK1 reporter plasmids‐induced luciferase activities within BXPC‐3 cells was analyzed by luciferase reporter gene assay (*n* = 3, ***p < 0.001).

For better understanding possible mechanism related to *MIR‐766‐3P*'s regulatory effect on PC tumorigenesis, a *RNA‐seq experiments* was performed (Figure 4a). Combining the target prediction databases miRtarbase, TarBase, and targetscan8.0 databases with screened differentially expressed genes, 135 candidate genes which might be regulated by *MIR‐766‐3P* were identified (Figure 4b). Through reviewing the literatures, *MAPK1* was selected because of its critical tumorigenic effect on the regulation of tumor occurrence from all of these candidate genes. Meanwhile, significantly high expression of *MAPK1* in PC was observed after analyzing of TCGA and GTEX database (Figure 4c). Then, based on the level of *MAPK1* expression, we classified all the samples into low or high expression group. Clearly, *MAPK1* overexpression was obviously related to a dismal prognostic outcome (Figure 4d). Next, the possible binding sites in *MAPK1* with *MIR‐766‐3P* were predicted based on targetscan and predictions were verified by dual‐luciferase reporter assay. As a result, *MIR‐766‐3P* showed direct targeting relation with *MAPK1* at the 3'‐UTR region to exert its regulatory function. *MIR‐766‐3P* overexpression significantly inhibited WT‐*MAPK1*‐3'‐UTR luciferase activity, rather than MUT–*MAPK1*‐3'–UTR (Figure 4e,f).

**FIGURE 5:**
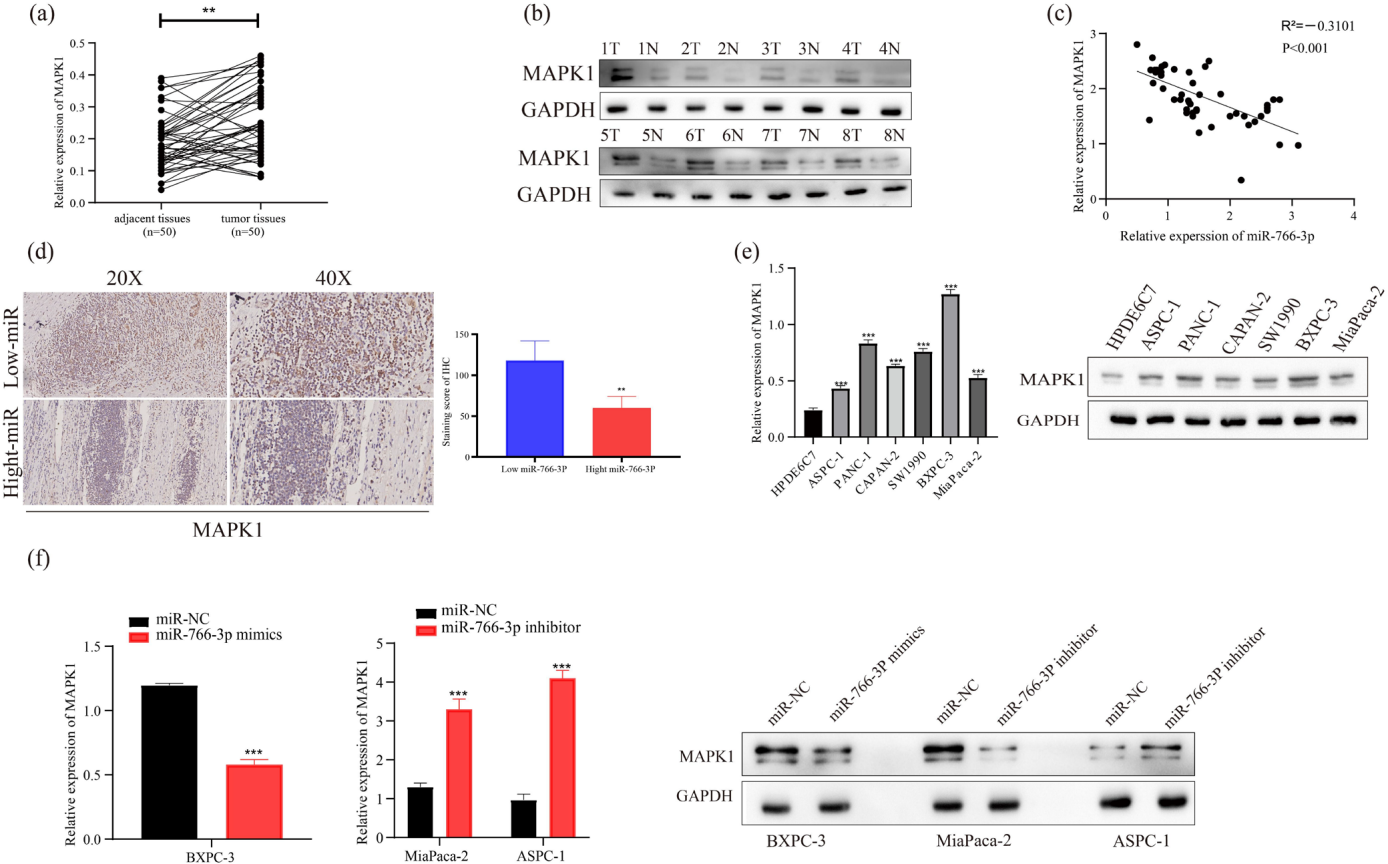
MAPK1 expression pattern in PC. (a, b) MAPK1 level markedly elevated within PC samples. (c) Correlation of MAPK1 level with miR‐766‐3p expression. (d) Immunohistochemistry showed that MAPK1 levels within PC samples were related to miR‐766‐3p expression. (e) MAPK1 mRNA and protein expression was upregulated within some PC cells. (f) MAPK1 levels within BXPC‐3, MiaPaca‐2, and ASPC‐1 cells after transfection were detected through WB and qRT‐PCR assays (*n* = 3, **p < 0.01, ***p < 0.001).

Subsequently, *MAPK1* mRNA and protein expression within PC and peritumoral tissues was measured through qRT‐PCR and WB, respectively. Relative to the peritumoral samples, tumor samples exhibited significantly elevated *MAPK1* expression (Figure 5a,b). Additionally, *MIR‐766‐3P* level was negatively related to *MAPK1* expression within tumor samples (Figure 5c). As confirmed by immunohistochemical analysis, cases showing *MIR‐766‐3P* downregulation have relatively high *MAPK1* expression (Figure 5d). We also measured the *MAPK1* mRNA and protein levels in PC cells. As a result, in comparison with controls, human PC cells exhibited significantly high *MAPK1* expression. Typically, *MAPK1* expression was highest in the BXPC‐3 cell line, which had a lower amount of *MIR‐766‐3P* expression. However, *MAPK1* expression decreased in two groups, while ASPC‐1 and MiaPaca‐2 had increased *MIR‐766‐3P* levels (Figure 5e). Meanwhile, following *MIR‐766‐3P* mimics as well as inhibitor transfection, respectively, cells were tested for *MAPK1* mRNA and protein expression, as a result, *MIR‐766‐3P* and *MAPK1* had a negative relationship (Figure 5f). All of the above results indicated that *MIR‐766‐3P* expression showed negative relation to *MAPK1*, while *MIR‐766‐3P* suppressed *MAPK1* by targeting the corresponding 3'‐UTR.

### 3.4 MIR‐766‐3P Suppresses the Growth, Spheroid Formation, and Cell Cycle Progression by MAPK1

**FIGURE 6:**
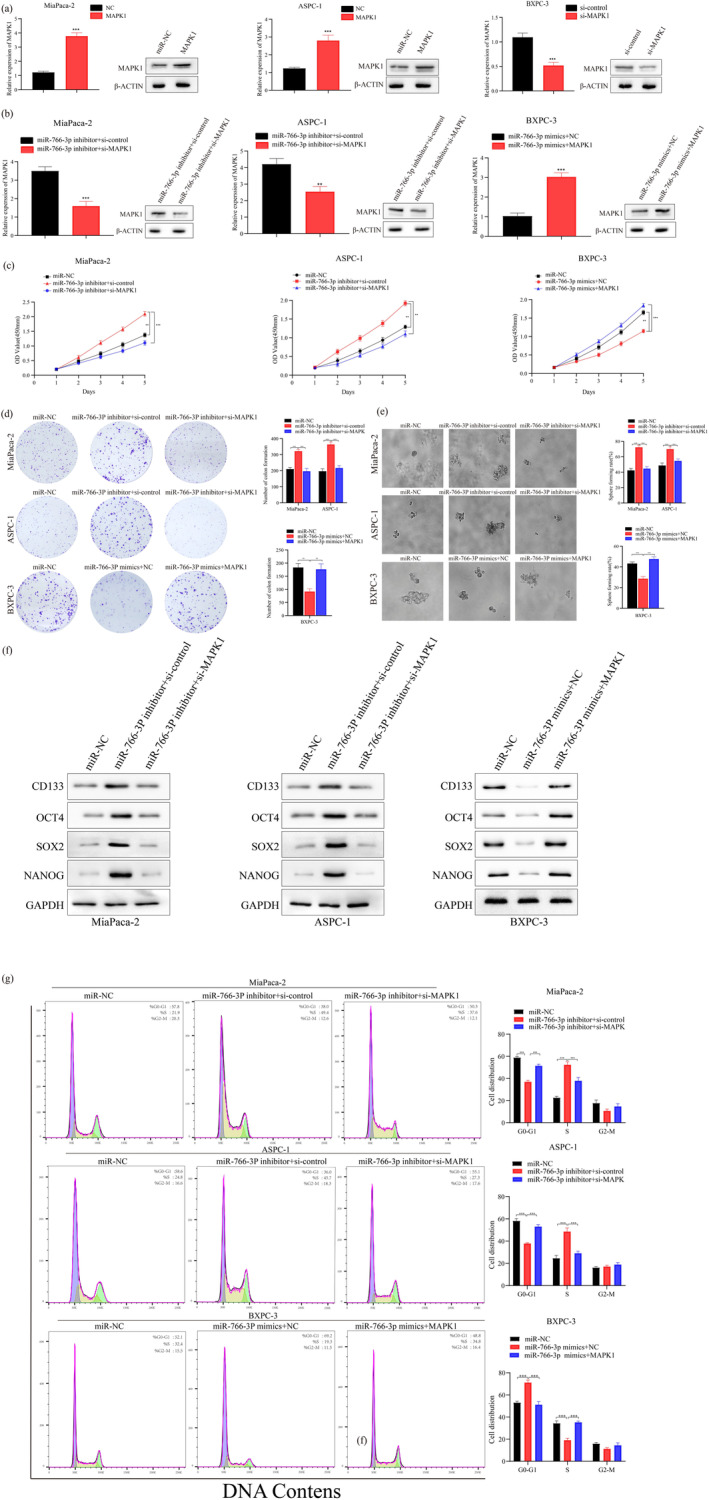
miR‐766‐3p regulated cell growth, spheroid forming ability as well as cell cycle via targeting MAPK1. (a) MAPK1 levels were confirmed within those transfected PC cells through WB and QRT‐PCR assays. (b) MAPK1 levels were verified in CO‐transfected PC cell lines through WB and QRT‐PCR assays. (c, d) CCK‐8 and clone‐forming assays exhibited the growth of CO‐transfected PC cell lines. (e, f) Spheroid forming rate (100 μm) and stemness‐associated gene expression within CO‐transfected cell lines. (g) Cell cycle changes of PC cells after CO transfection (*n* = 3, **p < 0.01, ***p < 0.001).

For investigating relationship between *MIR‐766‐3P* and *MAPK1* within PC, a rescue assay was designed. Three sets of cell lines were compared twice. The first comparison was between the cells after cotransfection of *MIR‐766‐3P* mimics + NC with *MIR‐766‐3P* mimics + *MAPK1*. The second comparison was between the cells subject to *MIR‐766‐3P* inhibitor + si‐control transfection and those subject to *MIR‐766‐3P* inhibitor + si‐*MAPK1* transfection. In the MiaPaca‐2, ASPC‐1 cell lines, we upregulated *MAPK1* using pcdna3.1‐*MAPK1*. In BXPC‐3 cells, we downregulated *MAPK1* using sirna‐*MAPK1*. *MAPK1* levels were analyzed through qRT‐PCR and WB assays (Figure 6a). In the MiaPaca‐2, ASPC‐1 cells, the *MIR‐766‐3P* inhibitor + si‐*MAPK1* cotransfection suppressed the expression of *MAPK1* compared with those of cells subject to *MIR‐766‐3P* inhibitor + si‐control cotransfection. However, the BXPC‐3 cells subject to *MIR‐766‐3P* mimics + *MAPK1* cotransfection exhibited higher *MAPK1* expression than that of the cells subject to *MIR‐766‐3P* mimics + NC cotransfection (Figure 6b).

Subsequently, *MAPK1* downregulation remarkably abolished *MIR‐766‐3P* silencing's promotion on MiaPaca‐2 and ASPC‐1 cell growth. Similarly, upregulation of *MAPK1* also attenuated the inhibitory effect of *MIR‐766‐3P* overexpression on proliferative capacity of BXPC‐3 cells (Figure 6c,d). Next, we examined the effect of *MAPK1* on the stemness sphere‐forming ability within MiaPaca‐2, ASPC‐1, and BXPC‐3 cells, as a result, for the former two cell lines, the promoted cell stemness sphere‐forming ability after *MIR‐766‐3P* inhibition could be abolished by the downregulation of *MAPK1*. While *MAPK1* upregulation abrogated *MIR‐766‐3P* overexpression's inhibition against stemness sphere formation ability. The WB assay was performed to analyze stemness markers (*CD133*, *OCT4*, *SOX2*, and *NANOG*), the results of which also suggested the effect of *MAPK1* on counteracting *MIR‐766‐3P*'s function in cell stemness ability (Figure 6e,f). For exploring *MAPK1*'s effect on mediating *MIR‐766‐3P*'s impact on the cell cycle, FCM detection was conducted. As a result, upregulation and downregulation of *MAPK1* abolished the *MIR‐766‐3P* overexpression or inhibition‐induced impacts on the cell cycle progression respectively (Figure 6g).

In summary, the above results confirmed that *MIR‐766‐3P* can exert effects on PC carcinogenesis by targeting *MAPK1*. And *MAPK1* can rescue the effects resulting from *MIR‐766‐3P* upregulation or inhibition on cells.

### 3.5 Effect of MIR‐766‐3P on the Progression of PC

**FIGURE 7:**
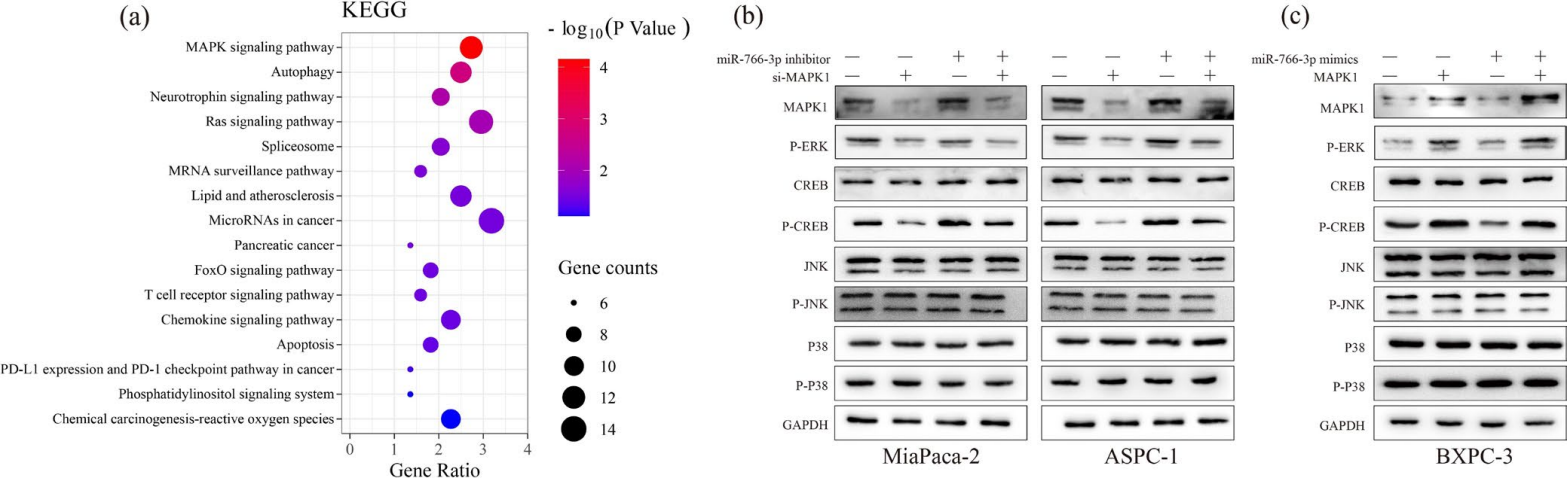
miR‐766‐3P regulated the MAPK/ERK pathway via MAPK1 within PC. (a) KEGG and GO analysis on those major related pathways. (b) Effects of mir‐766‐3p silencing plus MAPK1 knockdown on ERK/CREB/JNK/p38 together with the corresponding phosphorylation expression within MiaPaca‐2 and ASPC‐1 cells. (c) Effects of mir‐766‐3p overexpression plus MAPK1 upregulation on ERK/CREB/JNK/p38 together with the corresponding phosphorylation expression within BXPC‐3 cells (*n* = 3).

According to Figure 7a, the aberrant *MIR‐766‐3P* level was significantly enriched mainly in the *MAPK/ERK* pathways. (Figure 7a). *MAPK1* represents a critical biomarker for the *MAPK/ERK* pathway, which is associated with tumor occurrence and is an important step during tumor occurrence (Jung et al. 2016; Wu et al. 2016). *MAPK/ERK* is similarly involved in PC development and progression, which represents an essential pathway for regulating PC progression. Previously, the *MAPK/ERK* signaling pathway has an important effect on proliferation, migration, invasion, EMT, and cell cycle of PC cells (Sheng et al. 2017; Zhang et al. 2020). Therefore, our study focused on the role of *MIR‐766‐3P* in regulating the malignant phenotypes and contributes to a poor prognosis of PC through the *MAPK1* and *MAPK/ERK* pathway. According to our results, *MIR‐766‐3P* inhibition remarkably enhanced *ERK/CREB* phosphorylation, but *MAPK* knockdown effectively restored this promotive effect. Similarly, *MIR‐766‐3P* upregulation decreased *ERK/CREB* phosphorylation, while the upregulation of *MAPK1* similarly counteracted inhibition of *MIR‐766‐3P* overexpression against *ERK/CREB* phosphorylation. However, *MIR‐766‐3P* expression did not affect *JNK/P‐38* phosphorylation (Figure 7b,c), suggesting the role of *MIR‐766‐3P* in activating *MAPK/ERK* pathway for executing the biological activities by targeting *MAPK1*.

### 3.6 MIR‐766‐3P Inhibits In Vivo PC Tumorigenesis and Progression

**FIGURE 8:**
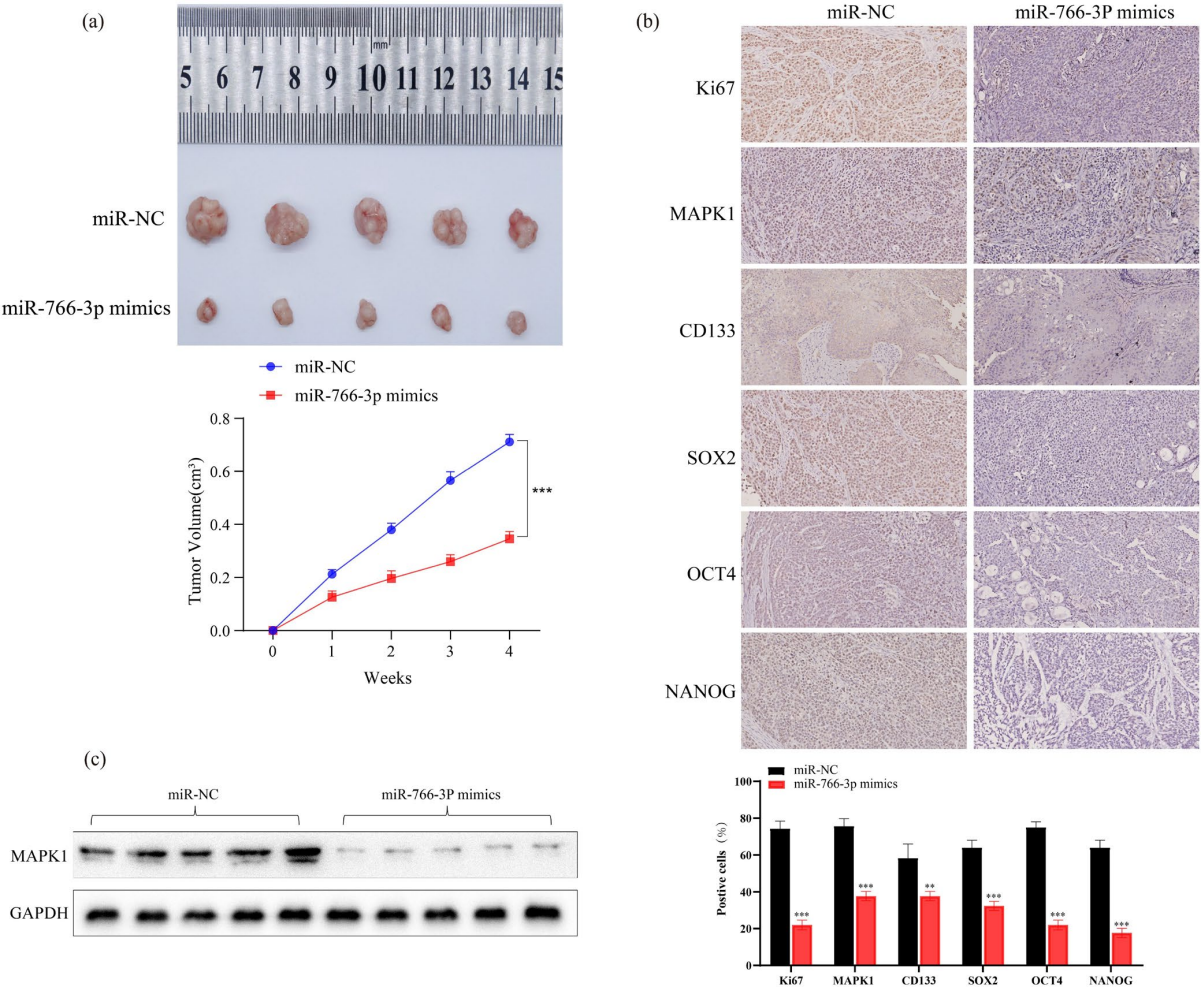
miR‐766‐3p inhibits ‐xenograft tumor development. (a) For mice given injection of miR‐766‐3P overexpression BXPC‐3 cells, tumor weight markedly decreased relative to those given injection of control cells (*n* = 5). (b) IHC assay was conducted for detecting Ki67, MAPK1, and stemness‐related gene levels within miR‐766‐3P and miR‐Ctrl tumors(X20). (c) According to WB assay, miR‐766‐3p mimics group had decreased MAPK1 levels of (*n* = 3, **p < 0.01, ***p < 0.001).

For investigating *MIR‐766‐3P*'s effect on in vivo tumorigenicity, the PC cells subject to control or *MIR‐766‐3P* mimics transfection were injected in the flank of each nude mouse subcutaneously. In comparison with the those given injection of control cells, mice subject to injection of *MIR‐766‐3P* mimics‐transfected cells exhibited small volume of tumors. And the tumor growth rate was also significantly reduced (Figure 8a). Additionally, compared with the mice injected with control cells, the mice injected with *MIR‐766‐3P* mimics‐transfected cells showed significantly low *MAPK1* expression in their tumor tissues (Figure 8b,c). And the expressions of tumor proliferation markers *Ki67* and tumor stemness markers (*NANOG*, *CD133*, *OCT4*, and *SOX2*) all obviously reduced compared with the controls (Figure 8d). Based on the above results, *MIR‐766‐3P* suppressed cancer progression in vivo.

## 4 Discussion

PC is a commonly identified cancer in the clinic. In the past period, PC treatment has clearly advanced with the improved surgical techniques and the addition of neoadjuvant and adjuvant treatments. However, PC still shows an increasing morbidity worldwide (Torphy, Fujiwara, and Schulick 2020). Because PC does not show early symptoms, often progresses to the middle or late stages by the time of diagnosis. Because PC is extremely malignant, there are no effective treatment options available in the clinic, thus resulting in a poor overall prognosis. PC has a low survival rate and remains relatively unchanged since the 1960s (Ansari et al. 2016) Therefore, it is urgently needed to identify effective ways for PC treatment.

Recently, miRNAs have been found to regulate tumor genesis and development, while miRNAs can regulate various genes and pathways associated with tumor occurrence and progression, thereby serving as tumor suppressor genes or oncogenes. Abnormal miRNAs levels within PC are often associated with PC pathogenesis (Xia, Chen, and Zhang 2021), and the different miRNA expressions influence the progression of PC, which may be the candidate biomarkers, therapeutic targets, and prognostic biomarkers (Daoud et al. 2019). The inhibitors and mimics of miRNA, which are being developed in preclinical trials, may be promising new regimens for cancer treatment (Lu and Rothenberg 2018; Li et al. 2014; Paladini et al. 2016).

*MIR‐766‐3P* is previously demonstrated to be the suppressor of multiple tumors, including liver cancer, renal cancer, and colorectal cancer. But the function in PC remains largely unclear. According to our results, *MIR‐766‐3P* level within PC cells and tissues markedly decreased relative to peritumoral tissues and normal pancreatic cells. Consequently, *MIR‐766‐3P*'s effects in vivo and in vitro were analyzed. According to our results, upregulation of *MIR‐766‐3P* inhibited growth, promoted arrest of cell cycle at G0/G1 phase, while dramatically decreasing in vivo and in vitro stemness sphere‐forming ability in PC cells. By contrast, *MIR‐766‐3P* silencing showed opposite effects, with significantly increased proliferation and stemness sphere‐forming ability within PC cells. Besides, relationship between the *MIR‐766‐3P* level and PC prognostic outcome was analyzed. As a result, high‐*MIR‐766‐3P*‐expressed group had a markedly increased survival rate compared to low‐*MIR‐766‐3P*‐expressed group. Together with the above in vivo and in vitro experimental analyses, *MIR‐766‐3P* has a critical effect in inhibiting pancreatic carcinogenesis.

In previous experiments, public databases were used to predict the targets of *MIR‐766‐3P*. For investigating related mechanisms by which *MIR‐766‐3P* affected PC, a RNA sequencing was used in combination with free databases for target prediction, and this approach exhibited higher reliability. Through the analysis, *MAPK1* was identified as a *MIR‐766‐3P* target. As an important pro‐oncogenic factor, *MAPK1* is highly expressed in multiple cancers (Xu et al. 2021; Xiong et al. 2021; Yu et al. 2023). And *MAPK1* also serves as an important biomarker for *MAPK/ERK* pathway. Activating *MAPK* signaling pathway has an important effect on the development of malignant phenotype in PC (Ikeda et al. 2012). Several published studies have shown that the mutations in *BRAF*, *KRAS*, and epigenetic dusp6 abrogation, could promote *MAPK* activation. By blocking the function of these genes, the malignant phenotype of PC can be attenuated (Furukawa 2015). The present work was the first to discover that *MIR‐766‐3P* exerted its regulatory function in PC by regulating *MAPK1*. By examining *MIR‐766‐3P* and *MAPK1* levels within PC cells and tissues, we found a negative association between them. And *MAPK1* reversed *MIR‐766‐3P*'s impact on cancer suppression. Therefore, *MIR‐766‐3P* directly affected *MAPK1* expression in PC cells, as evidenced through luciferase reporter, gain/loss of function, and correlation analysis. Furthermore, through KEGG pathway enrichment analysis and Genomes analysis, *MIR‐766‐3P* targeting *MAPK1* modulated pancreatic cell proliferation and controlled cell cycle through *MAPK/ERK* pathway, thereby affecting PC biological behavior. Subsequent experiments also validated this notion. The phosphorylation of *ERK/CREB* changed with the overexpression or inhibition of *MIR‐766‐3P*, and its level of phosphorylation could be subsequently reversed by the upregulation or downregulation of *MAPK1*.

## 5 Conclusion

In conclusion, this work analyzed *MIR‐766‐3P*'s role as well as the underlying mechanism on suppressing the malignant phenotype of PC, as well as the tumorigenesis and progression. Mechanically, *MIR‐766‐3P* impeded PC cell development, stem maintenance, and arrested cell cycle by directly targeting *MAPK1* and suppressing its expression, thus inhibiting the activation of *MAPK/ERK* pathway. Such results deepen our understanding of the effects of miRNAs on PC tumorigenesis and mechanism. And provided more sufficient research on *MIR‐766‐3P*'s role in PC, *MIR‐766‐3P* may become the novel marker for the prediction of the prognosis and PC treatment.

## Author Contributions

Zhipeng Quan and Ziwei Yin: investigation, writing – reviewing and editing. Yuelin Huang and Xuemei Huang and Hao Huang: data curation, writing – original draft preparation. Jianhua Gong: visualization. Qingrong Mo: supervision. Lingyun Liu: software, validation. Yi Zhou and Yaqun Yu: design, conceptualization and methodology. Zhipeng Quan, Ziwei Yin, Yuelin Huang contributed equally to this work and are co‐first authors. Yi Zhou and Yaqun Yu are co‐corresponding authors. All authors read and approved the final manuscript.

## Ethics Statement

The study has been approved by the Ethics Committee of the Affiliated Hospital of Guilin Medical University (grant number KJTLL202140), and the approval date is September 13, 2021. All participants in the study have provided appropriate informed consent as required by the ethics committee. The animal experiments have been approved by the Laboratory Animal Ethics Committee of Guilin Medical University (grant number GLMC202105162), and the approval date is May 16, 2021.

## Conflicts of Interest

The authors declare no conflicts of interest.

## Supporting information


Data S1.

Table S1.

Table S2.

## Data Availability

Data will be available upon reasonable requests from the corresponding author.
