# Supplementary material for: MiR‐766‐3p Inhibit the Proliferation, Stemness, and Cell Cycle of Pancreatic Cancer Cells Through the MAPK/ERK Signaling Pathway
Source: Mol Genet Genomic Med. 2024 Dec 18;12(12):e70049. doi: 10.1002/mgg3.70049 (PMC11653159; doi:10.1002/mgg3.70049)
Supplement: Supplementary file 1 — Data S1. [file MGG3-12-e70049-s001.docx]

>1.hsa-miR-766-sponge-1

GAATTCgaggagtggcctcacagctagtgtgcctgaattggcatgcctgtgagaggagcttgcatgtgagtgttttgtgttacatgcgctgtgatgggaaagagggtgctatcaaatatacacagaggattgcttagcctgcctgccaccgaacaactatgtcaaaagtccccagagtcttcacacaagccgtggtgtatgaagctgctgaggcGGTTgggctggagtGGGCCCgctgaggcGACTgggctggagtGGTACCgctgaggcTACCgggctggagtGCCGGCgctgaggcCGATgggctggagttggattggaagaagcggaagaaatctgttgcctgagtggagaagactgtacaatgtcctgtctctcaccatgggggatttttgtggcaggaaatgagagtaatgaggtccctgccaccaccttgaattatgggtggctccagaaaaccttattgtagattgagggggaagagaagggagagcgaggtcttgttgcatgctCTGTGCCTTCTAGTTGCCAGCCATCTGTTGTTTGCCCCTCCCCCGTGCCTTCCTTGACCCTGGAAGGTGCCACTCCCACTGTCCTTTCCTAATAAAATGAGGAAATTGCATCGCATTGTCTGAGTAGGTGTCATTCTATTCTGGGGGGTGGGGTGGGGCAGGACAGCAAGGGGGAGGATTGGGAAGACAATAGCAGGCATGCTGGGGATGCGGTGGGCTCTATGGGGATCC

>2.hsa-miR-766-sponge-2

GAATTCgaggagtggcctcacagctagtgtgcctgaattggcatgcctgtgagaggagcttgcatgtgagtgttttgtgttacatgcgctgtgatgggaaagagggtgctatcaaatatacacagaggattgcttagcctgcctgccaccgaacaactatgtcaaaagtccccagagtcttcacacaagccgtggtgtatgaagctgctgaggcTTGGgggctggagtGGGCCCgctgaggcTCGAgggctggagtGGTACCgctgaggcAATTgggctggagtGCCGGCgctgaggcGATAgggctggagttggattggaagaagcggaagaaatctgttgcctgagtggagaagactgtacaatgtcctgtctctcaccatgggggatttttgtggcaggaaatgagagtaatgaggtccctgccaccaccttgaattatgggtggctccagaaaaccttattgtagattgagggggaagagaagggagagcgaggtcttgttgcatgctCTGTGCCTTCTAGTTGCCAGCCATCTGTTGTTTGCCCCTCCCCCGTGCCTTCCTTGACCCTGGAAGGTGCCACTCCCACTGTCCTTTCCTAATAAAATGAGGAAATTGCATCGCATTGTCTGAGTAGGTGTCATTCTATTCTGGGGGGTGGGGTGGGGCAGGACAGCAAGGGGGAGGATTGGGAAGACAATAGCAGGCATGCTGGGGATGCGGTGGGCTCTATGGGGATCC
