# Supplementary material for: MiR‐766‐3p Inhibit the Proliferation, Stemness, and Cell Cycle of Pancreatic Cancer Cells Through the MAPK/ERK Signaling Pathway
Source: Mol Genet Genomic Med. 2024 Dec 18;12(12):e70049. doi: 10.1002/mgg3.70049 (PMC11653159; doi:10.1002/mgg3.70049)
Supplement: Supplementary file 2 — Table S1. [file MGG3-12-e70049-s002.docx]

**Table S1 Antibody information**

**Antibody name Lot number WB Concentration Producers**

| 60004-1-lg  81115-1-RR  66666-1-Ig  11263-1-AP  11064-1-AP  ab109250  51068-1-AP  28733-1-AP  [AF6188](http://www.affbiotech.cn/goods-1819-AF6188-CREB_Antibody.html)  [AF3189](http://www.affbiotech.cn/goods-1367-AF3189-Phospho_CREB_Ser133_Antibody.html)  [AF6318](http://www.affbiotech.cn/goods-1925-AF6318-JNK1_2_3_Antibody.html)  [AF3318](http://www.affbiotech.cn/goods-1483-AF3318-Phospho_JNK1_2_3_Thr183_Tyr185_Antibody.html)  14064-1-AP  28796-1-AP  27309-1-AP |
| --- |

| GAPDH  β-Actin  CD133  OCT4  SOX2  NANOG  MAPK1  P-ERK  CREB  P-CREB  JNK  P-JNK  P38  P-P38  Ki67 |
| --- |
|  |

| 1:5000  1:5000  1:2000  1:1000  1:1000  1:5000  1:2000  1:5000  1:2000  1:2000  1:2000  1:2000  1:2000  1:2000  1:3000 |  |  |  |  |
| --- | --- | --- | --- | --- |

| Proteintech  proteintech proteintech proteintech proteintech  Abcam  proteintech  proteintech  Affinity  Affinity  Affinity  Affinity  proteintech  proteintech  proteintech |
| --- |

**Antibody name Lot number IHC Concentration Producers**

| 66666-1-Ig  11263-1-AP  11064-1-AP  ab109250  51068-1-AP  27309-1-AP |
| --- |

| CD133  OCT4  SOX2  NANOG  MAPK1  Ki67 |
| --- |

| 1:100  1:150  1:100  1:200  1:500  1:3000 |  |  |  |  |
| --- | --- | --- | --- | --- |

| proteintech proteintech proteintech  Abcam  proteintech  proteintech |
| --- |
