# Supplementary material for: MiR‐766‐3p Inhibit the Proliferation, Stemness, and Cell Cycle of Pancreatic Cancer Cells Through the MAPK/ERK Signaling Pathway
Source: Mol Genet Genomic Med. 2024 Dec 18;12(12):e70049. doi: 10.1002/mgg3.70049 (PMC11653159; doi:10.1002/mgg3.70049)
Supplement: Supplementary file 3 — Table S2. [file MGG3-12-e70049-s003.docx]

Table S2 Sequence List

Sequence

| Forward  Reverse  Forward  Reverse  Forward  Reverse  Forward  Reverse |
| --- |

| MicroRNA-766-3p  MAPK1  U6  GAPDH |
| --- |

| 5′-ACTCCAGCCCCACAGCCT-3′  5′-TATTCGCACTGGATACGACCTGGAG-3′  5'-AACAGGCTCTGGCCCACCCA-3′  5'-AGTCCTCTGAGCCCTTGTCCTGA -3′  5′-CTCGCTTCGGCAGCACA-3′  5′-CTCGCTTCGGCAGCACA-3′  5′-ACAACTTTGGTATCGTGGAAGG-3′  5′-GCCATCACGCCACAGTTTC-3′ |
| --- |
